# SARS-CoV-2 Antibodies Mediate Complement and Cellular Driven Inflammation

**DOI:** 10.3389/fimmu.2021.767981

**Published:** 2021-11-02

**Authors:** Ida Jarlhelt, Sif Kaas Nielsen, Camilla Xenia Holtermann Jahn, Cecilie Bo Hansen, Laura Pérez-Alós, Anne Rosbjerg, Rafael Bayarri-Olmos, Mikkel-Ole Skjoedt, Peter Garred

**Affiliations:** ^1^ Laboratory of Molecular Medicine, Department of Clinical Immunology, Section 7631, Rigshospitalet, Copenhagen University Hospital, Copenhagen, Denmark; ^2^ Recombinant Protein and Antibody Laboratory, Department of Clinical Immunology, Section 7631, Rigshospitalet, Copenhagen University Hospital, Copenhagen, Denmark; ^3^ Institute of Immunology and Microbiology, University of Copenhagen, Copenhagen, Denmark

**Keywords:** SARS-CoV-2, antibody response, complement, monocytes, spike, receptor binding domain

## Abstract

The ongoing pandemic of coronavirus disease 2019 (COVID-19) caused by the severe acute respiratory syndrome coronavirus 2 (SARS-CoV-2) continues to constitute a serious public health threat worldwide. Protective antibody-mediated viral neutralization in response to SARS-CoV-2 infection has been firmly characterized. Where the effects of the antibody response are generally considered to be beneficial, an important biological question regarding potential negative outcomes of a SARS-CoV-2 antibody response has yet to be answered. We determined the distribution of IgG subclasses and complement activation levels in plasma from convalescent individuals using in-house developed ELISAs. The IgG response towards SARS-CoV-2 receptor-binding domain (RBD) after natural infection appeared to be mainly driven by IgG1 and IgG3 subclasses, which are the main ligands for C1q mediated classical complement pathway activation. The deposition of the complement components C4b, C3bc, and TCC as a consequence of SARS-CoV-2 specific antibodies were depending primarily on the SARS-CoV-2 RBD and significantly correlated with both IgG levels and disease severity, indicating that individuals with high levels of IgG and/or severe disease, might have a more prominent complement activation during viral infection. Finally, freshly isolated monocytes and a monocyte cell line (THP-1) were used to address the cellular mediated inflammatory response as a consequence of Fc-gamma receptor engagement by SARS-CoV-2 specific antibodies. Monocytic Fc gamma receptor charging resulted in a significant rise in the secretion of the pro-inflammatory cytokine TNF-α. Our results indicate that SARS-CoV-2 antibodies might drive significant inflammatory responses through the classical complement pathway and *via* cellular immune-complex activation that could have negative consequences during COVID-19 disease. We found that increased classical complement activation was highly associated to COVID-19 disease severity. The combination of antibody-mediated complement activation and subsequent cellular priming could constitute a significant risk of exacerbating COVID-19 severity.

## Introduction

Coronavirus disease 2019 (COVID-19) constitutes a global pandemic, caused by severe acute respiratory syndrome coronavirus-2 (SARS-CoV-2). COVID-19 related morbidity and mortality have been attributed to an exaggerated immune response and the ambiguous role of the complement system and its contribution to illness severity is being increasingly recognized (1). The complement system constitutes an essential part of innate immunity and the proteolytic cascade is initiated by three distinct pathways; the classical-, the lectin- and the alternative pathway. Each pathway differs on the mode of initiation, but all converge at the level of complement factor C3 to generate central and terminal effector molecules (2). The classical pathway can be initiated by the binding of C1q directly to the Fc portion of antibodies bound to antigens. The lectin pathway is initiated by binding of the so-called collectins and ficolins to carbohydrates directly on pathogens. Finally, the alternative pathway functions as an amplification loop for the classical and lectin pathway or might to some degree be initiated spontaneously when activated by C3 hydrolysis (3). Proper activation of the complement system will result in the elimination of the pathogen through effector functions including opsonization, pro-inflammatory secreted cleavage products, and microbial lysis by the terminal complement complex (TCC) (4). While complement may effectively contribute to the control of viral infection, activation of the complement pathways may also contribute to several pathologies observed in severe COVID-19 patients, due to its potent proinflammatory effects (5).

Several studies have suggested that the complement system is implicated in the pathogenesis of COVID-19 (6–8). It is generally accepted that the complement system plays a central role in acute respiratory distress syndrome (ARDS), which is also typically seen in severely COVID-19 disease (9). In a previous study regarding SARS-CoV-1, which is closely related to SARS-CoV-2, it was found that the activation of complement component C3 can aggravate the disease in SARS-CoV-associated ARDS (10). Moreover, *in vitro* studies have suggested that the spike protein of SARS-CoV-2 activates the alternative pathway (11) and that the nucleocapsid (N) protein from SARS-CoV-1, MERS-CoV and SARS-CoV-2 binds to MASP-2, which is the central serine protease in the lectin pathway (12). This binding results in increased lectin pathway-mediated complement activation and exacerbates inflammatory lung damage. Blockade of the N-protein:MASP-2 interaction or complement inhibition reduces hyperactivation of the complement system and lung damage. In addition, patients with age-related macular degeneration (AMD), in which exaggerated complement activation plays a central role in the etiology, have been shown to have a significantly increased risk of adverse clinical outcomes following SARS-CoV-2 infection (13). Conversely, a group of patients with genetic backgrounds of complement deficiency had no need for mechanical ventilation and they overcame their illness with less complications (13). Together, these data suggest that an exaggerated complement activation level predispose individuals to adverse outcomes associated with SARS-CoV-2 infection. Additionally, several studies have demonstrated an accumulation of activated complement proteins in damaged tissues and organs (14, 15) as well as elevated plasma levels of C5a and soluble TCC in infected patients (7, 16, 17). It has been suggested that a blockade of C5a may be crucial for inhibition of the cytokine storm and, therefore, would be a potential therapeutic target for acute lung damage caused by pathogenic viral infections, such as SARS-CoV-1 and -2 (18). At the time being, several approaches are conceivable to control complement activation as a therapeutic principle in COVID-19 (19).

More data are emerging showing the involvement of especially the lectin and alternative pathway of complement in COVID-19 pathology and the beneficial antibody-mediated viral neutralization in response to SARS-CoV-2 infection has been firmly characterized. We have approached some of the potential adverse outcomes of a SARS-CoV-2 antibody response and the linkage to an exacerbated immune response through the classical pathway. We hypothesized that the developed SARS-CoV-2 antibodies might contribute to enhanced complement and cellular-driven inflammation, exacerbating COVID-19 disease.

## Materials and Methods

### Buffers

The following buffers were used: PBS (10.1 mM Na_2_HPO_4_, 1.5 mM KH_2_PO_4_, 137 mM NaCl, 2.7 mM KCl), PBS-Tween (PBS-T) [PBS, 0.05% Tween-20 (8221840050, Merck)], PBS-T-EDTA [PBS, 0.05% Tween-20, 5 mM EDTA (EDS-500G, Merck) and Barbital-Tween (Barbital-T) [4 mM C_8_H_11_N_2_NaO_3_, 145 mM NaCl, 2.6 mM CaCl_2_, 2.1 mM MgCl_2_, 0.05% Tween-20].

### Plasma From Convalescent and Healthy Individuals

A total of 180 recovered individuals previously tested RT-PCR-positive for SARS-CoV-2 were included in the study. The Department of Emergency Medicine at Herlev University Hospital in Denmark recruited the participants and EDTA plasma samples were stored in aliquots and kept frozen at −80°C until used. The RT-PCR positive participants are comprised of males and females aged from 18–86 and the course of disease ranged from mild to severe based on an electronic self-report questionnaire. Mild disease was defined as having few symptoms and generally feeling well, moderate disease as being bedridden at home, and severe disease as the need for hospitalization. Based on previous measurements of IgG levels (20), individuals could further be divided into groups based on IgG levels as followed: high IgG, intermediate IgG and low IgG. A total of 60 EDTA plasma samples collected from healthy blood donors before 2020, having no SARS-CoV-2 antibodies, were used as negative controls.

### Measurements of IgG Subclasses by Sandwich ELISA

Nunc™ MaxiSorp Flat-Bottom 96-Well plates (442404, Thermo Fisher Scientific) were coated with 1 µg/ml RBD in PBS overnight (ON) at 4°C. The RBD antigen was produced as previously described (20). Plates were blocked with PBS-T for 1 h. Samples were diluted 1:50 in PBS-T-EDTA, applied to the plates in a 3-fold dilution and incubated for 1 h at room temperature (RT). HRP-conjugated monoclonal antibodies against IgG1 (A10648, Thermo Fisher Scientific). IgG2 (ab99779, abcam), IgG3 (05-3620, Thermo Fisher Scientific) and IgG4 (A10654, Thermo Fisher Scientific) were applied in a concentration of 1 µg/ml in PBS-T and incubated for 1 h RT. TMB ONE (4380A, KemEnTec Diagnostics) was used as a substrate and allowed to react for 10 minutes. The reaction was stopped with 0.3 M H_2_SO_4_ and the optical density (OD) of the samples was measured at 450-630 nm using A Synergy HT absorbance reader (BioTek Instruments). Plates were washed three times with PBS-T between the steps mentioned above. The data is presented in signal to noise (S/N) ratios between the sample OD value and a negative quality control.

An ELISA control experiment of the detection of each IgG subtypes were performed in which normal human immunoglobulin (ZLB Behring) or Bovine Serum Albumin (BSA) (Sigma-Aldrich) were coated in a 2-fold dilution, starting in 10 µg/ml incubating ON at 4°C. Each of the 4 subclass antibodies was used for detection in a concentration of 1 µg/ml. TMB ONE and 0.3 M H_2_SO_4_ were used for revealing as described above.

### Measurements of Complement Deposition by Sandwich ELISA

Nunc™ MaxiSorp Flat-Bottom 96-Well plates (442404, Thermo Fisher Scientific) were coated with 3 µg/ml RBD in PBS ON at 4°C. Plates were blocked with PBS-T for 1 h. Samples were diluted 1:60 (for high IgG samples) and 1:20 (for intermediate, low and negative IgG samples) in PBS-T-EDTA, applied to the plates in a 3-fold dilution and incubated for 1 h at RT. A normal human serum pool was applied in a 1:50 dilution and incubated for 45 min at 37°C. Biotinylated monoclonal antibodies against C4b (Hyb 162-02, Bioporto Diagnostics), C3bc [in-house produced, clone BH6 (21)] and TCC [in-house produced, clone aE11 (22)] were applied in a concentration of 2 µg/ml in PBS-T and incubated for 1 h RT. Streptavidin-HRP conjugate (Sigma-Aldrich) was added to the wells for 1 h in a 1:2500 dilution in PBS-T. TMB ONE (4380A, KemEnTec Diagnostics) was used as a substrate and allowed to react for 8 minutes. The reaction was stopped with 0.3 M H_2_SO_4_ and the OD of the samples was measured at 450-630 nm using a Synergy HT absorbance reader (BioTek Instruments). Plates were washed three times with PBS-T between the steps mentioned above.

### Depletion of Antibodies Against RBD

Nunc™ MaxiSorp Flat-Bottom 96-Well plates (442404, Thermo Fisher Scientific) were used to deplete antibodies against RBD by coating with 10 µg/ml RBD or BSA in PBS ON at 4°C. Plates were blocked with PBS-T for 1 h. EDTA plasma from three convalescent individuals with high levels of IgG and healthy controls were each transferred to a RBD and BSA coated well in a 1:50 dilution and incubated for 30 min at RT shaking. The samples were transferred to the next RBD or BSA coated well and once again incubated for 30 min at RT shaking. This was repeated through 12 wells along with the ELISA plate and the sample was left in the final well ON at 4°C. To ensure that each sample was depleted for antibodies against RBD, the samples was analyzed in a new plate coated with either RBD or spike protein (1 µg/ml), and HRP-conjugated polyclonal rabbit antibodies against human IgM (P0215), IgA (P0216), or IgG (P0214) (all from Agilent Technologies) in a concentration of 1 µg/ml was used to detect the remaining antibodies bound to each of the antigens. The depleted samples were afterwards subjected to the complement deposition assay as described above and a neutralization assay as described elsewhere (23). The principle of the neutralization assay is to address the neutralizing capacity of the developed antibodies based on the interaction between recombinant human ACE-2 ectodomain and the SARS-CoV-2 RBD. Briefly, Nunc MaxiSorp microtiter plates (442404, Thermo Fisher Scientific) were coated with 1 μg/ml of ACE-2 overnight at 4°C in PBS. The following day, 2% EDTA plasma/mAbs were incubated for 1 h in low-binding round-bottom plates (Thermo Fisher Scientific) with a solution of biotinylated RBD (4 ng/ml) with HS-strep-HRP (1:16,000 dilution) in PBS-T. Afterwards, the mAbs:RBD:HS-Strep-HRP solution was transferred to ACE-2 plates and incubated for 15 min. The plates were developed with TMB One (4380A, KemEnTec Diagnostics) for 20 min and stopped with 0.3 M H_2_SO_4_. The absorbance was read at 450-630 nm. Between steps, the plates were washed twice with PBS-T.

### Cytokine Production in Human Monocytes

A human THP-1 monocytic cell line (88081201, Sigma-Aldrich) and human monocytes isolated from a healthy blood donor were used to address the cellular mediated inflammatory response due to Fc-gamma receptor (FcγR) engagement by SARS-CoV-2 specific antibodies. MagniSort™ Human CD14 Positive Selection Kit (8802-6834-74, Invitrogen) was used for the isolation of human monocytes according to the manufacturer’s instructions. TPP^®^ tissue culture 6-well plates (Z707767, Sigma-Aldrich) were coated with 10 µg/ml RBD or LPS and incubated ON at 4°C. Heat-inactivated sera from a pool of individuals previously infected with SARS-CoV-2 were applied in a 1:50 dilution and incubated for 2h at RT. Plates were washed with sterile PBS and THP-1 cells or freshly isolated monocytes were applied in a total concentration of 1x10^6^ cells for each condition/in each well. Non-treated cells and cells incubated with fluid-phase LPS were included as controls. The cells were incubated for 24 hours in a humidified atmosphere at 37°C and 5% CO_2_. Cytokine release within the resulting supernatants was analyzed by the Luminex^®^ 100/200™ System (Invitrogen), using a custom-made Bio-Plex Pro™ Human Cytokine Panel (#12011278, Biorad). The following cytokines were measured: IL-6, IL-1β and TNF-α. Analyses were performed according to the manufacturer’s instructions (Instruction Manual #10000092045). All work was performed under sterile conditions.

### Statistics

Statistical analyses were performed using GraphPad Prism version 9.0.0 (GraphPad Software, CA, USA) and R (version 3.6.1 for Windows, R Foundation for Statistical Computing, Vienna, Austria). Estimation of levels of deposited C4, C3 and TCC were interpolated by regression analysis using a four-parameter logistic curve fitting, and results were given in arbitrary units/ml (AU/ml). In a 1:50 dilution, the calibrator was defined to contain 200 AU/ml. Non-parametric data were log-transformed before performing statistical analysis. Statistical differences between disease severity and IgG levels groups were analyzed using Kruskal–Wallis test with a Tukey’s multiple comparison test. Multiple regression models were used to assess the relationship between complement activation and IgG subclass levels and the independent variables of disease severity, age and IgG levels groups. Spearman rank correlation tests were used to determine the correlation between antibody levels and complement activation products. An ordinary one-way ANOVA with Holm-Šídák’s multiple comparisons test was used to determine the statistical differences between groups in the neutralization- and deposition assay post depletion of RBD antibodies, as well for the differences between cytokine release of monocytes under different conditions. Significance levels are as follows: *p < 0.05, **p < 0.01, ***p < 0.001, ****p < 0.0001; *p* < 0.05 was considered statistically significant.

## Results

### Levels of IgG1, IgG2, IgG3 and IgG4 in Convalescent Plasma

**Figure 1:**
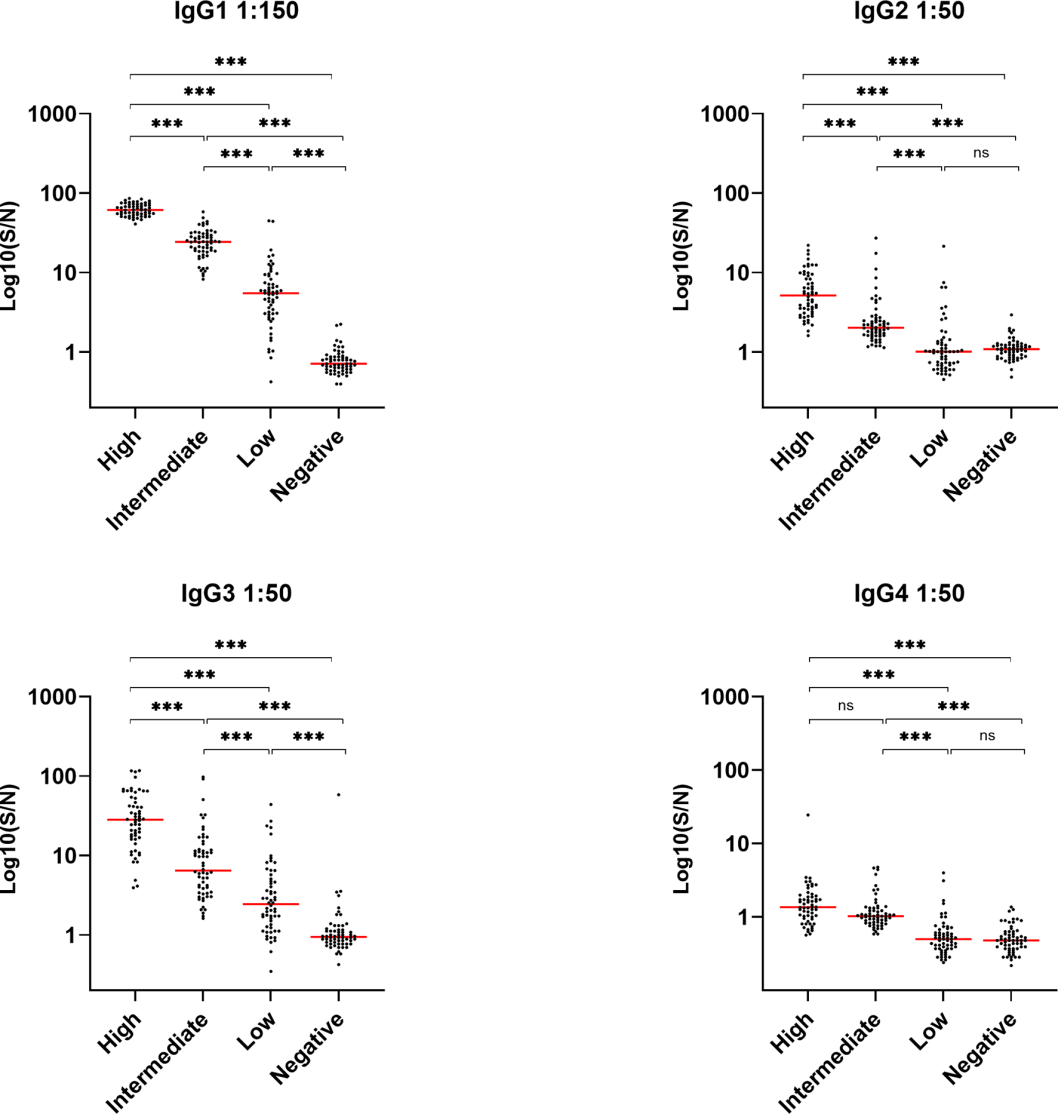
Detection of IgG1, IgG2, IgG3 and IgG4 in recovered SARS-CoV-2 individuals. Groups divided according to levels of total IgG; high, intermediate, low and negative healthy controls, n = 240. Levels were assessed by coating plates with 1 µg/nm RBD and detecting with HRP-conjugated antibodies against IgG1, 2, 3 and 4 (1 µg/ml). Samples were measured in a 1:50 dilution, except for IgG1 which were measured in a 1:150 dilution. Dynamic range represented in signal-to-noise ratios (S/N). A p value < 0.05 was considered significant. ns, not significant, ***p < 0.001 using Tukey all-pair comparisons.

The levels of IgG in the convalescent samples were previously determined in a study, where we showed that the IgG levels were significantly correlated to COVID-19 disease severity (20). In order to look further into the IgG response during COVID-19, we determined the distribution of IgG subclasses IgG1, -2, -3 and -4, in a cohort of previously infected individuals. The quantitative relative levels of the four subclass antibodies were measured in EDTA plasma from 240 individuals in total, with 60 individuals in each of four groups divided according to total IgG levels (i.e. high IgG, intermediate IgG, low IgG and no IgG/healthy controls. IgG1 was by far the most abundant subclass antibody, followed by prominent detection of IgG3 - both with significantly different levels between each of the four IgG groups. However, low levels of IgG2 and IgG4 were also detected (**Figure 1**). All subclass antibodies correlated significantly with the levels of IgG. Results from a control experiment validating that each commercial antibody correctly binds one IgG subtype, verified in an ELISA, are presented in **Supplementary Figure 1**.

### Complement Activation Mediated by SARS-CoV-2 Antibodies

**Figure 2:**
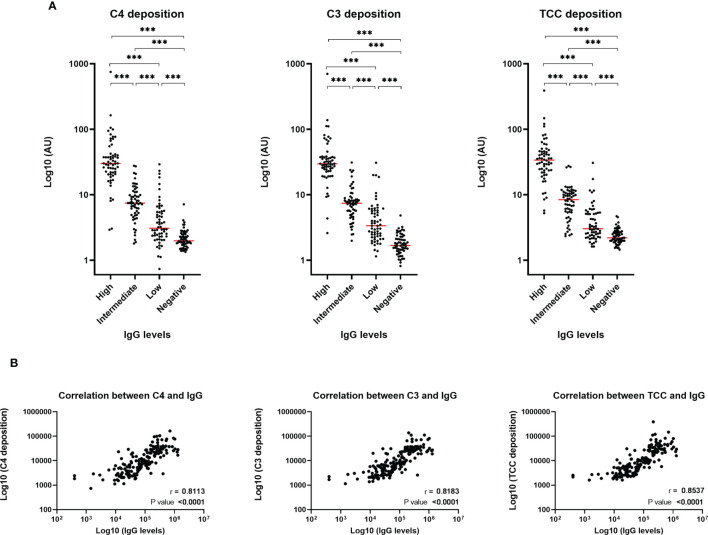
Complement deposition in recovered SARS-CoV-2 individuals. **(A)** Measurements of complement components C4, C3 and TCC in convalescent EDTA plasma. Groups divided according to levels of total IgG; high, intermediate, low and negative healthy controls, n = 240. Levels were assessed by coating plates with 3 µg/nm RBD, followed by incubation with EDTA plasma diluted 1:60 (for high IgG samples) and 1:20 (for intermediate, low and negative IgG samples). A normal human serum pool was applied in a 1:50 dilution. Biotinylated monoclonal antibodies against C4b, C3bc and TCC (2 µg/ml) was used for detection. Dynamic range represented in arbitrary units (AU). A p value < 0.05 was considered significant. ***p < 0.001 using Tukey all-pair comparisons. **(B)** The correlation between deposition of either C4, C3 or TCC and IgG levels using Spearman rank correlation analysis.

**Figure 3:**
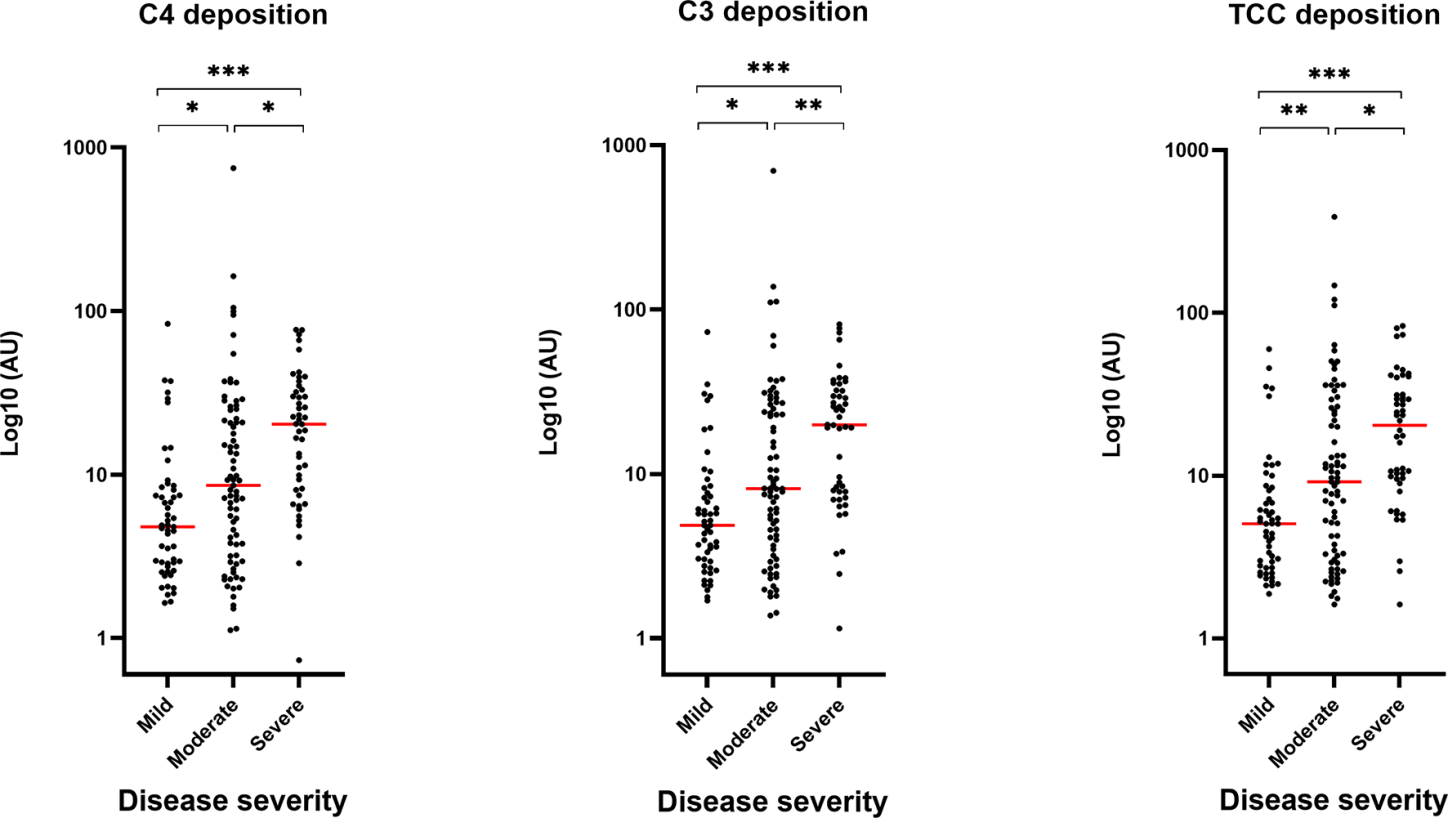
Correlation between complement deposition, IgG levels and severity. Groups divided according to disease severity. Dynamic range represented in arbitrary units (AU). A p value < 0.05 was considered significant. *p < 0.05, **p < 0.01, ***p < 0.001 using Tukey all-pair comparisons, n = 180.

To evaluate the role of the complement system during COVID-19 infection, we investigated if the developed antibodies against SARS-CoV-2 antigens would activate the classical pathway *in vitro*. The activation of complement on SARS-CoV-2 antigen/antibody complexes was measured in three setups detecting deposition of complement components C4, C3 and TCC and the data show that individuals with high levels of IgG will have equivalent high complement deposition (**Figure 2A**). A strong positive correlation (r > 0.8) were seen for IgG and the three complement components (**Figure 2B**). Deposition levels of complement components C4, C3 and TCC also correlated internally (**Supplementary Figure 2**). The deposition of complement did additionally correlate with disease severity, implying that plasma from individuals with either high levels of IgG and/or a more severe course of the disease, in general, would have increased complement activation through deposition of C4, C4 and TCC (**Figure 3**).

### Depletion of Antibodies Against RBD in Convalescent Plasma

**Figure 4:**
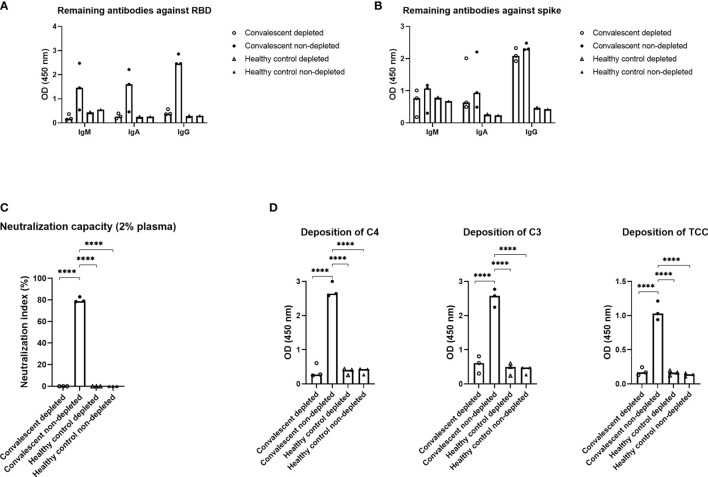
Effect of depletion of antibodies against RBD. Plasma from three recovered convalescent individuals and healthy control individuals was depleted for anti-RBD antibodies and subjected to the following assays; **(A)** an RBD specific sandwich ELISA, **(B)** a spike specific sandwich ELISA, **(C)** a neutralizing assay and **(D)** a complement deposition assay, with spike as the antigen. The RBD and spike specific assays utilized plates coated with RBD or spike (1 µg/ml) and HRP-conjugated polyclonal rabbit antibodies against human IgM, IgA, or IgG (1 µg/ml) for detection. A p value < 0.05 was considered significant. ****p < 0.0001 using an ordinary one-way-ANOVA with Holm-Šídák’s multiple comparisons test. Data are represented as mean values of two independent measurements.

To assess the importance of the RBD domain for complement activation, plasma from three individuals with high levels of IgG against SARS-CoV-2, along with three negative controls, were subjected to solid phase depletion of antibodies against RBD (**Figure 4**). The depleted and non-depleted samples were afterwards subjected to immobilized RBD (**Figure 4A**) or full-length spike (**Figure 4B**) to verify that the antibodies were in fact depleted. The resulting data show that all IgM, IgA and IgG antibodies against RBD are removed, while antibodies with epitopes on the remaining parts of spike are still present in the plasma samples. Neutralization capacity for the remaining antibodies (**Figure 4C**) and deposition of complement on spike (**Figure 4D**) was decreased significantly post RBD depletion, indicating that Ig directed towards this domain will play an essential role in complement activation.

### Activation of Human Monocytes by SARS-CoV-2 Immune Complexes

**Figure 5:**
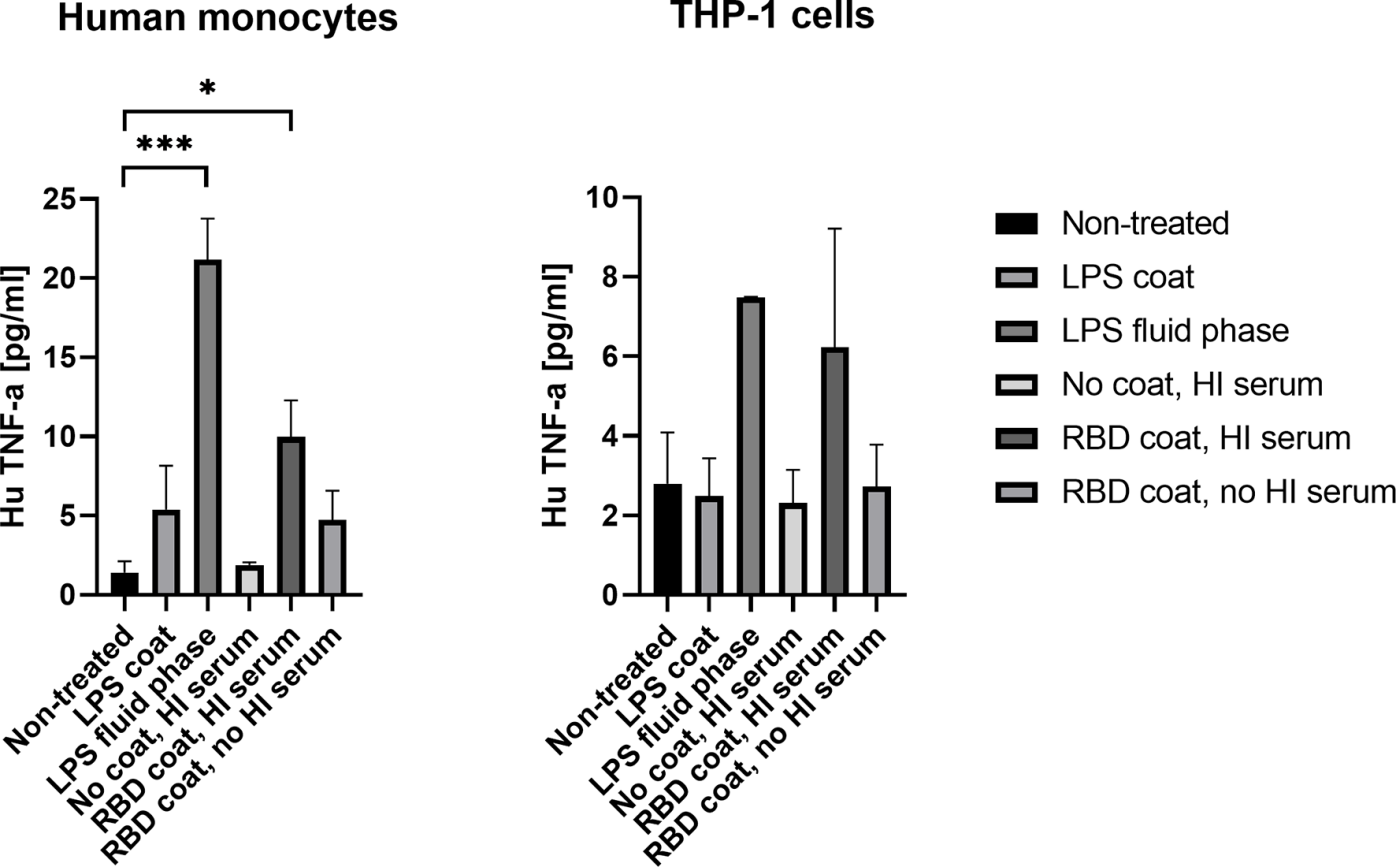
Production of cytokine TNF-α in isolated human monocytes and THP-1 cells after stimulation with SARS-CoV-2 immune complexes. A p value < 0.05 was considered significant. *p < 0.05, ***p < 0.001 using an ordinary one-way-ANOVA with Holm-Šídák’s multiple comparisons test.. Data are represented as mean values of three independent measurements.

To address the axis connecting humoral and cellular inflammation, we primed THP-1 monocytes and freshly isolated human monocytes with SARS-CoV-2 antigen/antibody immune complexes, the antigen being RBD. The resulting Fc gamma charging with anti RBD antibodies resulted in a significant rise in pro-inflammatory cytokine, TNF-α, in the freshly isolated human monocytes (*p* = 0.0194) (**Figure 5**). LPS was included as a positive control, both immobilized in the solid phase and directly added to the cells during incubation (fluid-phase), and the latter appeared to result in more activation of the monocytes. The same tendency was seen for the THP-1 monocytes, but the differences were not significant due to higher background activation of the cells (*p* = 0.1874). Priming with immune complexes did not give rise to increased production of cytokines IL-6 and IL-1β in either freshly isolated monocytes or THP-1 monocytes (**Supplementary Figure 3**).

## Discussion

A hallmark of severe COVID-19 disease is excessive inflammation associated with enhanced morbidity and mortality. In addition, accumulating evidence suggests that overactivation of the complement system plays a critical role in the pathophysiology and inflammatory response during severe COVID-19 disease (1). We hypothesized that anti-SARS-CoV-2 antibodies may contribute to exacerbated immune responses through classical complement pathway activation. Due to the polyclonal nature of antibodies, each subclass displays distinct functions and features and the distribution of the developed antibodies therefore becomes important to control viral infection, such as COVID-19. In line with the findings in recent reports (24–27) our results indicate that SARS-CoV-2 specific IgG1 and IgG3 were the dominant subclasses of IgG; while IgG2 and IgG4 were barely detected. IgG1 and IgG3 are known to efficiently trigger the classical complement pathway while IgG2 and IgG4 have reduced binding of C1q (28). Viral infections in general lead to IgG antibodies of the IgG1 and IgG3 subclasses, with IgG3 antibodies appearing first in the course of the infection (29). In a similar manner, there appears to be a preferential generation of IgG3 during COVID-19 infection. Our findings agree with another study where a substantial difference in the spike-specific IgG subclass composition was observed. A larger proportion of S1 and RBD-specific IgG3 was associated with COVID-19 severity (30). The study shows that spike-specific IgG1, and not IgG3, was most closely correlated with *in vitro* viral neutralization, leading the authors to conclude that excess IgG3 may play an inflammatory role in the pathogenesis of COVID-19 in some individuals. This somehow unbalanced IgG response, enriched in IgG3, may promote immunopathology and excess inflammation rather than tissue repair, exacerbating the symptoms of COVID-19 patients. Furthermore, our study showed that complement activation through deposition of C4, C3 and TCC appears to be mediated by the developed antibodies against SARS-CoV-2. More specifically, this seems to occur through the antibodies binding to epitopes on RBD, since the complement deposition was almost completely absent post depletion of RBD antibodies in convalescent plasma samples using spike protein as a target for complement activation. The exact residues of RBD domain that are involved in triggering the complement activation through interaction with the produced antibodies are unknown. However, in a recent publication epitope binning experiments were performed and revealed several epitope hotspots within the RBD (23). Whether these are equally important for complement activation needs to be further studied. A meta-analysis has suggested that RBD specific antibodies are the most dominant in COVID-19 patients (27). In addition, recent studies have reported that deceased COVID-19 patients have more protein N-specific antibody responses while the convalescent response is mainly driven by spike specific antibodies, suggesting the antigen-specific antibodies influence the immunity effectiveness and disease development (31). However, in terms of inflammatory contribution have different modes of action from the developed antibodies been reported. A recent publication indicates that human N protein targeting antibodies might inhibit excessive complement activation (32). The authors reported a COVID-19 patient-derived human monoclonal antibody targeting the N protein, which upon binding to the SARS-CoV-2 antigen in an *ex vivo* assay, were able to avoid hyperactivation of complement in patients recovering quickly.

Our findings indicate that the levels of complement activation products are positively correlated with both IgG levels and disease severity. Although C3 and TCC measurements reached slightly different significance levels between severity groups, the same tendency is seen for all three complement components. Of notice we see the most pronounced spreading/distribution in the moderate group. The findings agree with a recent study conducting a systematic review and meta-analysis, investigating possible differences in the serum concentrations of the complement components, C4 and C3, in COVID-19 patients with different severity and survival status (33). The conclusion was that both C4 and C3 levels were significantly lower in patients with high disease severity or non-survivor status than patients with low severity or survivor status, indicating that C4 and C3 are more readily consumed in patients with a severe or fatal course of the disease. Another study found that the complement system was one of the intracellular pathways most highly induced by SARS-CoV-2 infection in lung epithelial cells (34). The genes whose transcription was most highly induced by SARS-CoV-2 was encoding C1 proteases C1R and C1S, CFB, and complement C3. The authors conclude that the induction of complement expression and C3 protein activation in airway epithelial cells is a SARS-CoV-2–driven event and not a bystander event triggered by exacerbated inflammation. Interestingly, this may be a mechanism common to pandemic coronaviruses, because mouse models of the related SARS-CoV-1 infection, have indicated that C3, C1R, and CFB are all part of a pathogenic gene signature correlating with lethality (35). One could speculate whether increased complement activation is a broad indicator of critical disease, or whether increased complement activation contributes to severe illness. In a recent publication they investigated complement activation in blood from patients with COVID-19 compared with two non-COVID cohorts: patients hospitalized with influenza and patients admitted to the intensive care unit with acute respiratory failure requiring invasive mechanical ventilation (36). They demonstrated that circulating markers of complement activation were elevated in patients with COVID-19 compared with those with influenza and to patients with non–COVID-19 respiratory failure. Moreover, the results indicated that enhanced activation of the alternative pathway was most prevalent in patients with severe COVID-19 and was associated with markers of endothelial injury and hypercoagulability; all together identifying complement activation as a distinctive and possibly pathogenic feature of COVID-19. In addition, it might be expected that increased antibody production and complement activation are a result of high viral load. However, a study demonstrated that total levels of IgG were not correlated with viral load in COVID-19 patients, while a weak negative correlation was observed between IgG subclasses and viral load (27). These findings are consistent with two prior studies, which also reported a missing correlation between persistent SARS-CoV-2 RNA and neutralizing antibody titers (37, 38). Considering the negative correlation between viral load and antibody levels, the role of IgG subclasses, especially IgG1 and IgG3, could be very relevant parameters during COVID-19 infection. Taken together, the findings give an essential insight into the immunological response during a COVID-19 infection and further research is needed into the potentially harmful effects of SARS-CoV-2 antibodies and their interaction with the complement system during infection.

Monocytes and monocyte-derived macrophages are professional phagocytes, utilizing Fc, complement, lectin- and scavenger receptors to facilitate endocytosis. They are also highly active biosynthetic and secretory cells, contributing to inflammation, immunity and antiviral responses (39). It has been demonstrated that myeloid cell recruitment to the lungs contributes to the hyperinflammatory state and coagulation during ARDS (40). COVID-19 infection is known to be accompanied by an aggressive inflammatory response with the release of a large amount of pro-inflammatory cytokines, also referred to as the “cytokine storm” (41, 42). In order to address the cellular mediated inflammatory response as a consequence of FcγR engagement by SARS-CoV-2 specific antibodies, we evaluated cytokine secretion of IL-6, IL-1β and TNF-α by THP-1 cells and freshly isolated monocytes after incubation with RBD immune complexes. Much of the research focus has been on the role of antibodies and FcγRs during viral infections in relation to virus neutralization and antibody-dependent enhancement of infection, while data on FcγR-mediated cytokine responses in the context of viral infections is limited and somehow conflicting (43). However, it has been suggested that anti-spike antiserum can induce hyperinflammation *via* macrophage FcγR, depending on IgG glycosylation and involving Syk, which is a tyrosine kinase involved in intracellular signaling (44). Our investigations indicate that FcγR stimulation of the monocytes by SARS-CoV-2 antibodies result in a significant increase in the production of TNF-α, but not IL-6 and IL-1β. It is nevertheless important to highlight that an induced cytokine profile depends on the collaboration between FcγRs and other danger sensing receptors. For example, cross-activation of FcγRIIa and TLRs is known to strongly amplify the production of several pro-inflammatory cytokines (45, 46). In our analysis, we measure the effect of FcγR activation alone. In addition, the freshly isolated monocytes seemed to be slightly activated (although not significantly) on immobilized RBD, without SARS-CoV-2 antibodies present – this is most likely explained by the fact that human monocytes have been suggested to have small amounts of ACE-2 on the surface (47). Finally, THP-1 cells are, despite of many similarities to human monocytes, known to have distinct cytokine profiles compared to human monocytes. The lack of IL-6 or IL-1β secretion from the THP-1 cells in our experiment, even after incubation with LPS, is in agreement with previous literature (48). Taken together, it could be speculated whether an exacerbated immune response and antibody production toward SARS-CoV-2 may supplement to the mechanisms causing hyperactivation of macrophages and monocytes, being a contributing factor to the deadly cytokine storm which is a hallmark of COVID-19 disease. In relation, exaggerated type I IFN responses have additionally been reported to be involved in hyperinflammation and contribute to the severe progression of COVID-19 (49).

In conclusion, our findings support the notion that antibodies against SARS-CoV-2 could represent a two-edged sword. It is known that particularly antibody-dependent cellular cytotoxicity and complement-dependent cellular cytotoxicity can drive harmful and systemic proinflammatory responses that can have severe pathophysiological consequences. Our investigations indicate that SARS-CoV-2 antibodies might drive significant inflammatory responses through the classical complement pathway and cellular immune-complex activation that could have negative consequences during COVID-19 disease. However, it is important to note that the data presented does not give direct evidence of classical pathway activation, since a contribution from the lectin pathway cannot be excluded. The combination of antibody-mediated complement activation and subsequent cellular priming could constitute a significant risk of exacerbating COVID-19 severity.

## Data Availability Statement

The raw data supporting the conclusions of this article will be made available by the authors, without undue reservation.

## Ethics Statement

The studies involving human participants were reviewed and approved by Regional Ethical Committee of the Capital Region of Denmark (H-20028627). The patients/participants provided their written informed consent to participate in this study.

## Author Contributions

IJ, SN, and CJ performed laboratory determinations and analyzed the data. RB-O, M-OS, and PG designed the study. IJ, SN, CJ, CH, LP-A, AR, RB-O, M-OS, and PG wrote the manuscript. All authors critically reviewed the manuscript. All authors contributed to the article and approved the submitted version.

## Funding

This work was financially supported by the Carlsberg Foundation (CF20-0045) and the Novo Nordisk Foundation (205A0063505).

## Conflict of Interest

The authors declare that the research was conducted in the absence of any commercial or financial relationships that could be construed as a potential conflict of interest.

## Publisher’s Note

All claims expressed in this article are solely those of the authors and do not necessarily represent those of their affiliated organizations, or those of the publisher, the editors and the reviewers. Any product that may be evaluated in this article, or claim that may be made by its manufacturer, is not guaranteed or endorsed by the publisher.

## Supplementary Material

Supplementary Figure 1 An ELISA control experiment of the detection of IgG1, -2, -3 and -4.

Supplementary Figure 2 Correlation between deposition of C4, C3 and TCC.

Supplementary Figure 3 Production of cytokines IL-6 and IL-1β in human monocytes and THP-1 cells after stimulation with SARS-CoV-2 immune complexes.
