# Supplementary figures and images for: SARS-CoV-2 Antibodies Mediate Complement and Cellular Driven Inflammation
Source: Front Immunol. 2021 Nov 2;12:767981. doi: 10.3389/fimmu.2021.767981 (PMC8596567; doi:10.3389/fimmu.2021.767981)

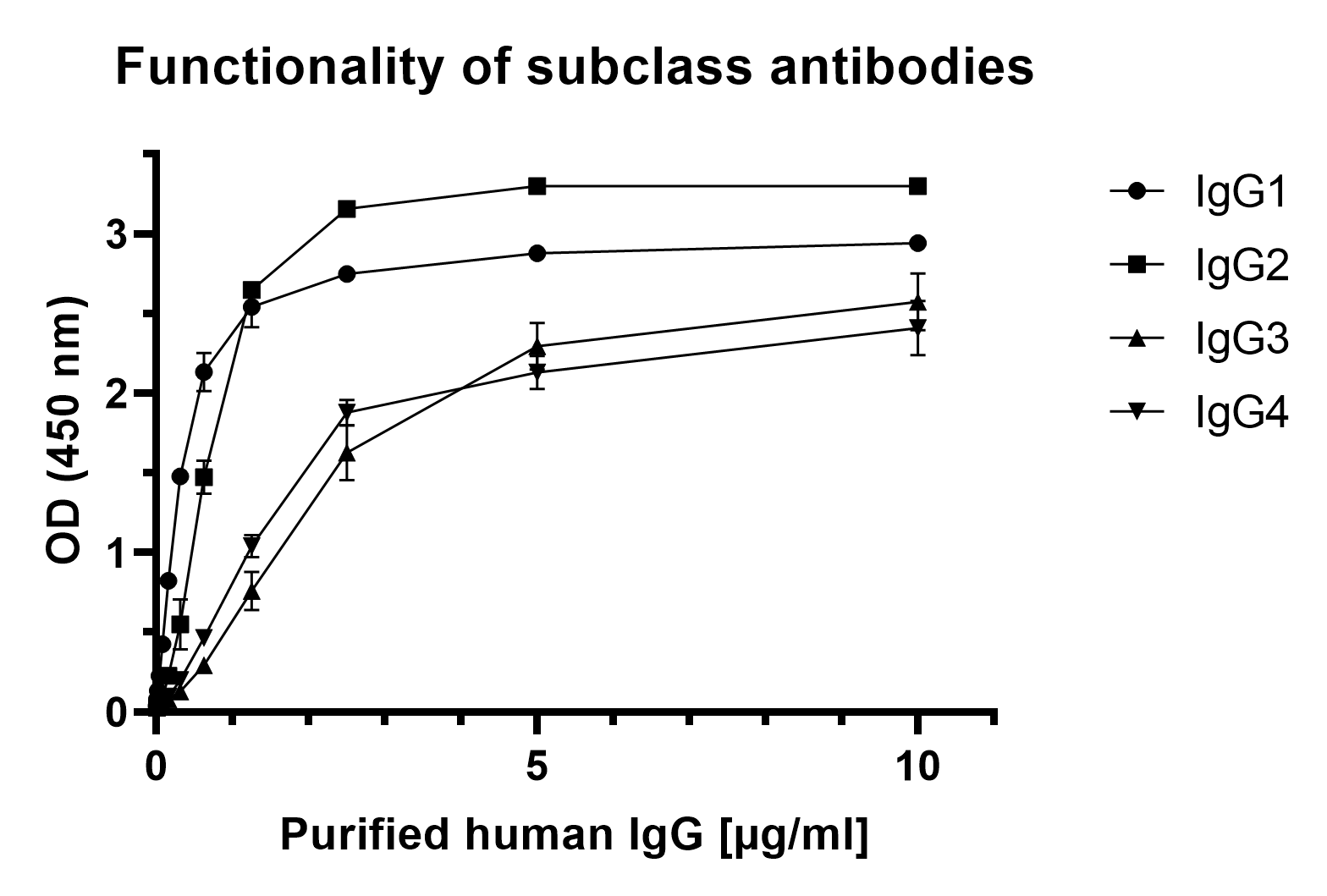

Supplement: Supplementary Figure 1 — An ELISA control experiment of the detection of IgG1, -2, -3 and -4. [file Image_1.tif]

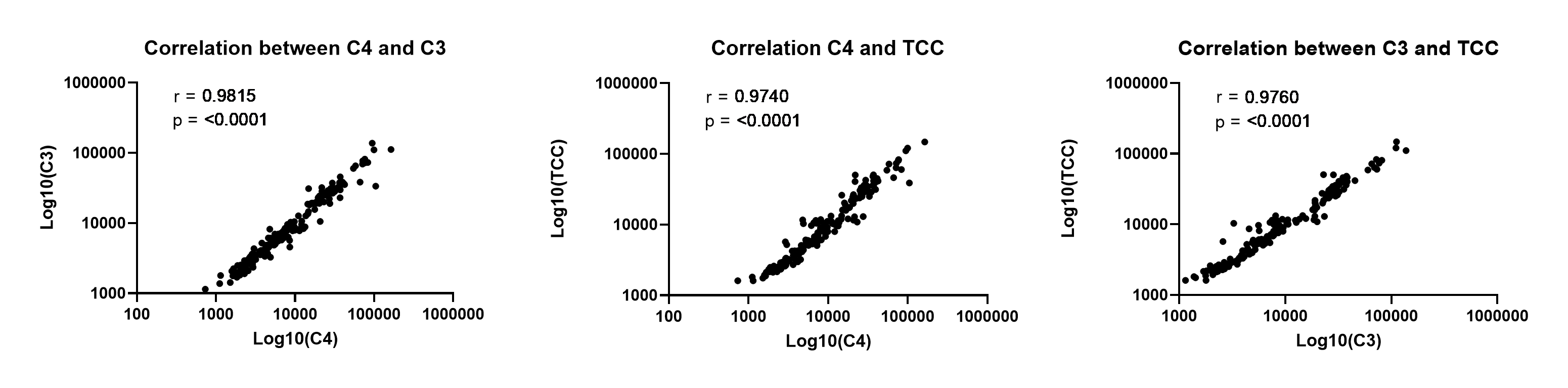

Supplement: Supplementary Figure 2 — Correlation between deposition of C4, C3 and TCC. [file Image_2.tif]

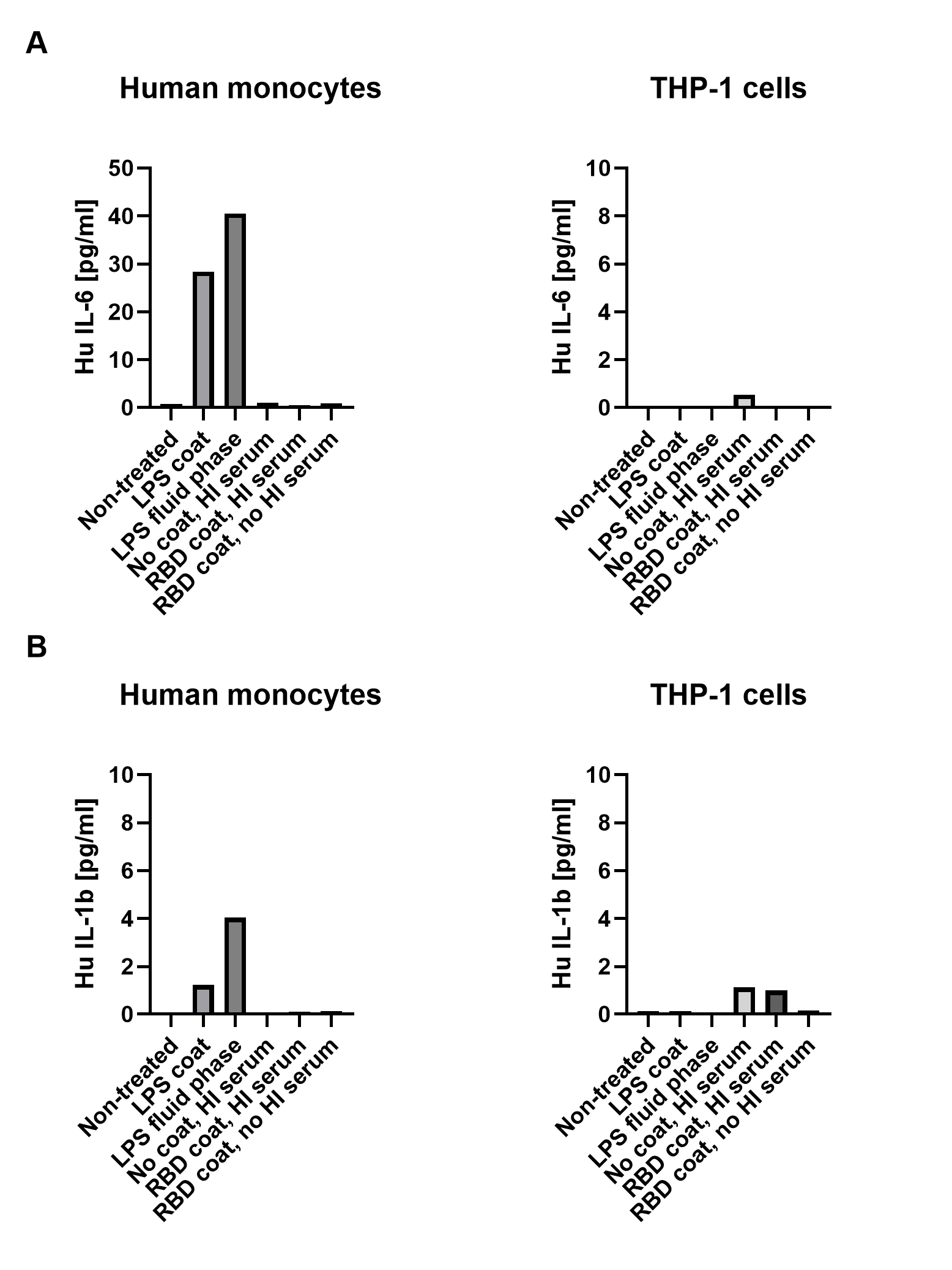

Supplement: Supplementary Figure 3 — Production of cytokines IL-6 and IL-1β in human monocytes and THP-1 cells after stimulation with SARS-CoV-2 immune complexes. [file Image_3.tif]
